# One-Pot Multicomponent Synthesis of Methoxybenzo[*h*]quinoline-3-carbonitrile Derivatives; Anti-Chagas, X-ray, and In Silico ADME/Tox Profiling Studies

**DOI:** 10.3390/molecules26226977

**Published:** 2021-11-19

**Authors:** Hegira Ramírez, Katiuska Charris, Esteban Fernandez-Moreira, Benjamín Nogueda-Torres, Mario V. Capparelli, Jorge Ángel, Jaime Charris

**Affiliations:** 1 Laboratorio de Síntesis Orgánica, Facultad de Farmacia, Universidad Central de Venezuela, Apartado 47206, Los Chaguaramos, Caracas 1041-A, Venezuela; katiuska.charris@gmail.com; 2 Facultad de Medicina, Universidad de Las Américas, Quito 170503, Ecuador; 3 Escuela de Medicina, Universidad Espíritu Santo, Samborondón 092301, Ecuador; esteban.f.moreira@gmail.com; 4 Escuela Nacional de Ciencias Biológicas, Departamento de Parasitología, Instituto Politécnico Nacional, Mexico City 11340, Mexico; bnogueda@yahoo.com; 5 Unidad de Estructura Molecular, Fundación Instituto de Estudios Avanzados (IDEA), Apartado 17606, Caracas 1015-A, Venezuela; mariocapparelli@hotmail.com; 6 Laboratorio de Síntesis Orgánica y Diseño de Fármacos, Dpto. de Química, Facultad Experimental de Ciencias, Universidad del Zulia, Maracaibo 4011-A, Venezuela; jangel63@yahoo.com

**Keywords:** cancer, chagas, one-pot synthesis, methoxybenzo[*h*]quinoline

## Abstract

Several methoxybenzo[*h*]quinoline-3-carbonitrile analogs were designed and synthesized in a repositioning approach to developing compounds with anti-prostate cancer and anti-Chagas disease properties. The compounds were synthesized through a sequential multicomponent reaction of aromatic aldehydes, malononitrile, and 1-tetralone in the presence of ammonium acetate and acetic acid (catalytic). The effect of the one-pot method on the generation of the target product has been studied. The compounds were in vitro screened against bloodstream trypomastigotes of *T. cruzi* (NINOA and INC-5 strains) and were most effective at showing a better activity profile than nifurtimox and benznidazole (reference drugs). A study in silico on absorption, distribution, metabolism, excretion, and toxicity (ADME/Tox) profiling to help describe the molecular properties related to the pharmacokinetic aspects in the human body of these compounds was reported. In addition, *X-ray* data for the compound 2-Amino-5,6-dihydro-4-(3-hydroxy-4-methoxy-phenyl)-8-methoxybenzo[*h*]quinoline-3-carbonitrile **6** was being reported. Spectral (IR, NMR, and elemental analyses) data on all final compounds were consistent with the proposed structures.

## 1. Introduction

Cancer and tropical diseases caused by protozoa are two dissimilar illnesses that manifest themselves through different symptoms. However, they share similar metabolic requirements related to a high proliferation rate [1]. Cancer is the second leading cause of death globally after ischemic heart disease and stroke. In 2018, there were an estimated 18.1 million new cases, and 9.6 million deaths from cancer, as per data registered by the World Health Organization (WHO) [2]. Prostate cancer (PCa) is the fifth most common cancer among men worldwide and the second most common cancer in the United States of America [3]. Although there are several types of cancer treatment options in practice, their success depends on the type and stage of cancer; however, they have limitations. To date, chemotherapy has played a central role in the clinical treatment of cancer, and numerous anticancer agents have been approved for this purpose [4,5,6,7].

On the other hand, parasitic infectious diseases due to pathogenic protozoans still constitute a major health problem worldwide and mainly occur in underdeveloped countries. One of them is Chagas disease (CD), also known as American trypanosomiasis, which is broadly dispersed in Latin America and the Caribbean, and affects between 6–7 million people with at least 12,000 dying each year [8,9]. It is a multisystemic disorder that can affect the cardiovascular, digestive, and central nervous systems. The CD is caused by *Trypanosoma cruzi*, a hemoflagellate parasite that is transmitted through various species of hematophagous reduviid insects (kissing bugs) mainly in endemic areas. Other routes of transmission include oral, transfusion, congenital, organ transplantation, and laboratory accidents [9].

Although chemotherapy is the mainstay of cancer therapy, most of the chemotherapy drugs cause general toxicity to any proliferating cells, which can severely limit the therapeutic value of these drugs [10]. In an attempt to overcome this problem, new anti-cancer agents with unique mechanisms of action have been developed; however, many of them have not been therapeutically useful due to low tumor selectivity. Treatment of CD can be divided into etiologic, that is, treating directly *T. cruzi* infection, or nonetiologic, treating the manifestations of the disease. There are only two therapeutic options for the treatment of the disease: nifurtimox (Nfx, Lampit™) and benznidazole (Bnz, Rochagan™), which were introduced into clinical therapy over six decades ago. In most cases these drugs are efficient in the acute phase of the disease, but unfortunately, they are almost ineffective in the chronic phase [8,9].

One approach to overcome this hurdle may be to develop a new use of existing drugs [11]. This repositioning or repurposing approach can be a very effective way to develop a new drug, since many existing drugs have been studied for their pharmacokinetics and safety profiles and often have already been approved by regulatory agencies for human use. An important facet of the repositioning approach is that the discovery of new targets can be parlayed directly into the generation of new chemical entities by structural analog derivatization that can further enhance the new mechanism or target activity. Therefore, we have applied a repositioning and lead optimization approach to developing effective anti-cancer and antimalarial agents using chloroquine (CQ) as a lead compound [12,13,14].

According to recent reports, it has been found that 2-amino-5,6-dihydro-4-phenylbenzo[*h*]quinoline-3-carbonitrile derivatives [15], 2-amino-4-benzylpyridine-3-carbonitrile derivatives [16], 2-amino-4-quinolinyl-naphthopyrane-3-carbonitrile derivatives [17], 6,7-Dimethyl-4-(3,4,5-trimethoxyphenyl)-3,4-dihydroquinolin-2(1H)-one (DTDQ) [18], and 2-Amino-4-(phenyl substituted)-*5H*-indeno[1,2-b]pyridine-3-carbonitrile derivatives [19] are particularly active as anticancer and MMPs inhibitors. This literature reveals that 1,2-dihydro naphthalene and *1H*-indene coupled heterocyclic compounds possess remarkable anticancer activity. On this basis, and as a continuation of previous work [15,19] we are reporting the synthesis, via a one-pot synthesis from elementary starting materials as methoxy substituted-1-tetralone, aromatic aldehydes substituted, malononitrile, ammonium acetate, acetic acid in toluene of 2-Amino-5,6-dihydro-4-(3 or 4-hydroxy-4 or 3-methoxyphenyl)-methoxybenzo[*h*]-quinoline-3-carbonitrile derivatives and their in vitro evaluation against bloodstream trypomastigotes of *T. cruzi* (NINOA and INC-5 strains). A study in silico on absorption, distribution, metabolism, excretion, and toxicity (ADME/Tox) profiling to help to describe the molecular properties related to the pharmacokinetic aspects in the human body of these compounds is reported. In addition, *X-ray* data for the compound 2-Amino-5,6-dihydro-4-(3-hydroxy-4-methoxy-phenyl)-8-methoxybenzo[*h*]quinoline-3-carbonitrile **6** is being reported.

## 2. Results and Discussion

### 2.1. Sinthesis of 2-Amino-5,6-dihydro-4-(3 or 4-hydroxy-4 or 3-methoxyphenyl)-methoxybenzo[h]-quinoline-3-carbonitrile Derivatives (***4***–***15***)

**Scheme 1:**
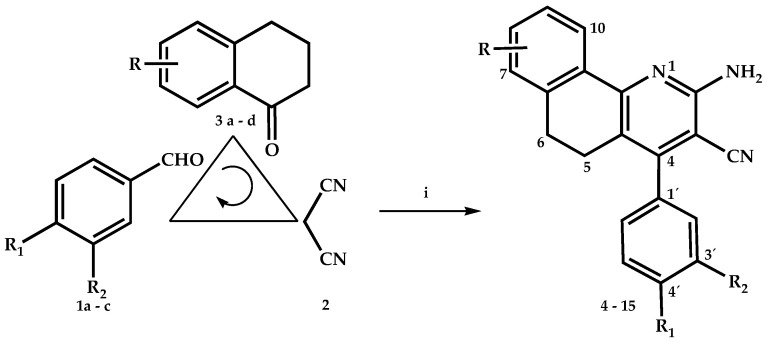
Multicomponent process for the synthesis of methoxybenzo[h]quinoline-3-carbonitrile derivatives **4**–**15**. **i**: Toluene, AcONH4, AcOH, Δ, 5 h.


**Scheme 2:**
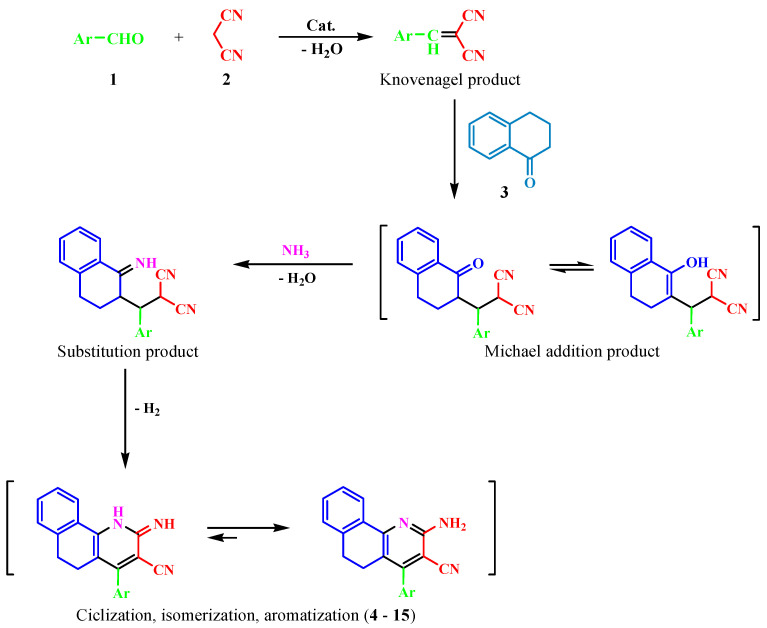
Proposed reaction mechanism for obtaining compounds **4**–**15**.

The synthesis of 2–aminobenzo[h]quinoline derivatives has been largely investigated; one of the most reported processes was related to a classic addition of Michael between malononitrile to 2 arylidene-1-tetralone or between benzylidenemalononitrile derivatives and 1-tetralone, respectively [15,20,21,22]. An environmentally-friendly and moderately efficient method for the preparation of 2–aminobenzo[h]quinoline derivatives were successfully developed via arylaldehyde, 1-tetralone, malononitrile, and ammonium acetate in ethanol by the “three-component” domino reaction under the thermal, microwave, and ultrasound methodologies [23,24]. It had further been reported that analog systems, such as 2-amino-4-aryl-3-cyanopyridines, indeno[1,2-b]pyridines could be constructed using a one-pot coupling reaction using microwave activation, or under solvent-free conditions and conventional heating mode [25,26]. However, the development of a general and efficient synthetic strategy to obtain 2–aminopyridine fused with a six-membered ring of the 1,2-dihydronaphthalene was still desired. As part of our ongoing program in this area [19], we now reported the preparation of a series of 2–amino-methoxybenzo[h]quinoline-3-carbonitriles in less time from the reaction and with better yields than reported previously [15,20,21,23,24], through the sequential multicomponent reaction of benzaldehyde substituted **1** (1 mmol), malononitrile **2** (1 mmol), 1-tetralone substituted **3** (1 mmol), and ammonium acetate (1.5 mmol) in toluene, with a catalytic amount of acetic acid, reflux, and the system equipped with a Dean-Stark trap (Scheme 1). The resultant reaction may be assumed to proceed via the Knoevenagel reaction with the formation of arylidenemalononitrile, which then underwent the Michael addition with 1-tetralone respective **3**, followed by cyclization, isomerization, and aromatization to obtain the final products **4**–**15** (Scheme 2). The analytical and spectral data of compounds **4**–**15** were consistent with their respective structures.

**No**

**R**

**R_1_**

**R_2_**

**Yield % ^a^**

**Yield % ^b^**

**4**
HOCH_3_OH8692
**5**
7-OCH_3_OCH_3_OH8189
**6**
8-OCH_3_OCH_3_OH8494
**7**
9-OCH_3_OCH_3_OH8291
**8**
HOHOCH_3_-93
**9**
7-OCH_3_OHOCH_3_-90
**10**
8-OCH_3_OHOCH_3_-91
**11**
9-OCH_3_OHOCH_3_-89
**12**
H

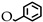

OCH_3_-83
**13**
7-OCH_3_

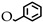

OCH_3_-87
**14**
8-OCH_3_

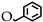

OCH_3_-80
**15**
9-OCH_3_

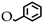

OCH_3_-84^a^: Stepwise yield % [15]; ^b^: Multicomponent process yield % for compounds **4**–**15**.

For the new compounds, **7**–**15,** the infrared (IR) spectra showed absorptions between 3444 and 3150 cm^−1^ assigned to the presence of −OH and −NH_2_. The presence of −CN group was confirmed by a stretching vibration between 2224 and 2208 cm^−1^, and a stretching vibration between 1616 and 1603 cm^−1^ indicated the presence of −N=C− of the heterocyclic group. In ^1^H NMR spectrum, two multiplets appeared around 2.59 and 2.66 ppm for H-5 and H-6, respectively, whereas protons of −OCH_3_ group appearred as a singlet (s) between 3.83 and 3.91 ppm in each compound; broad singles at 5.20 and 5.84 ppm confirmed the presence of protons of NH_2_ and OH, respectively, and the remaining aromatic protons of the 1,2-dihydronaphthalene and aldehydic moiety were reported according to the substitution pattern, respectively. The ^13^C NMR spectrum of the compounds exhibited peaks around 24, 28, 55, 56, 88, and 118 ppm for C_5_, C_6_, OCH_3_, C-CN, and −CN which were also confirmed by DEPT 135° and HETCOR experiments (see Supporting Materials). The analytical and spectroscopic data for all compounds were summarized in the experimental section.

**Figure 1:**
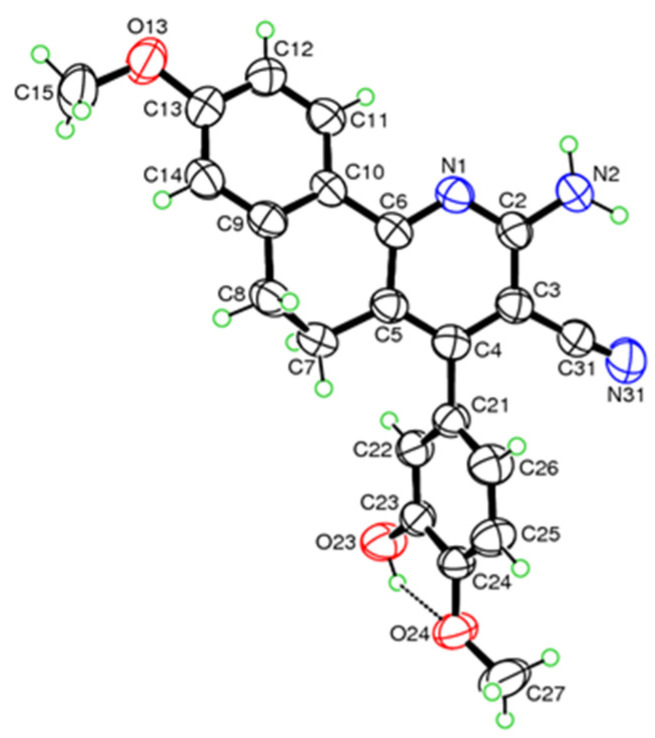
Molecular structure of compound **6** showing the atomic numbering. The displacement ellipsoids are drawn at 50% probability. A dashed line indicates an intramolecular hydrogen bond.

The X-crystal structure analysis for compound **6** showed that all the bond distances were within the expected values (Figure 1) [27]. In the tricyclic system, the central ring displayed an approximate screw-boat conformation [puckering parameters: q_2_ = 0.440(3) Å, q_3_ = −0.176(4) Å, *¦Õ*_2_ = 86.5(5)°, and Q = 0.474(3) Å)] [28]. The molecule formed an O-H···O(methoxy) intramolecular hydrogen bond. In addition, in the crystal structure, there were intermolecular hydrogen bonds of the types O-H···O(methoxy), O-H···N(cyano), N-H···O(methoxy), and N-H···O(hydroxyl), which linked the molecules to form a three-dimensional network, confirming the structure assignment. There was a disordered ethanol molecule of solvation close to a two-fold axis, which was given a 50% occupancy because both symmetry-related positions were mutually exclusive.

### 2.2. ADME/Tox Profile

The molecular structures of the synthesized 2–amino-methoxybenzo[h]quinoline-3-carbonitrile **4**–**15** were introduced in simplified molecular input line entry specification (SMILES) nomenclature into the ADME/Tox web tools SwissADME and pkCSM-pharmacokinetics programs, a free platform available online through the site (http://www.swissadme.ch/) and (http://structure.bioc.cam.ac.uk/pkcsm) (accessed on 30 September 2021), respectively [29,30,31]. We selected the most important ADME/Tox properties provided by the web tools to represent the ADME/Tox profile. According to the data obtained in the analysis (Table 1), absorption was predicted from water solubility, lipophilicity, and percentage of human intestinal absorption (HIA) properties. Water solubility was predicted using the Silicos IT LogSw descriptor of SwissADME. LogSw values for our compounds were predicted to range from −4.384 to −5.217. In the SwissADME LogSw scale, compounds with values less than −6 were considered to be poorly soluble, and additionally compounds **4**–**15** could be considered poorly soluble. Lipophilicity was assessed using the logarithm of the n-octanol/water partition coefficient, which was predicted using the Consensus LogPo/w descriptor of SwissADME. A general guide for good oral bioavailability (good permeability and solubility) was to have a moderate logP (log P < 5)[32,33]. For our compounds, the predicted values of logPo/w ranged from 3.27 to 4.84, and those of which could be considered moderate.

The percentages of the synthesized compounds **4**–**15** that would be absorbed through the human intestine (% HIA) were predicted using the web tool pkCSM-pharmacokinetics with a range from 94.97 to 100%, and therefore those values are considered appropriate.

The descriptors used to predict the distribution were the glycoprotein P (P-gp) substrate and fraction unbound (FU) descriptors, using web tool pkCSM-pharmacokinetics. All compounds were predicted to be substrates of P-gp. The fraction unbound influences renal glomerular filtration and hepatic metabolism and, consequently, affected the volume of distribution, total clearance, and efficacy of drugs [30,34]. The web tool pkCSM-pharmacokinetics allowed us to predict for compounds **4**–**15** that had low values on the order of 0.034 and 0.253 for the fraction unbound.

The excretion process occurred primarily as a combination of liver and kidney-mediated effects. This process was related to bioavailability and was important in determining dosage rates to achieve steady-state concentrations [30]. Excretion values were predicted, using the total clearance (CLtot) descriptor of web tool pkCSM-pharmacokinetics, to range from 0.434 to 0.570 mL/min/kg.

The toxicity levels of the synthesized compounds were predicted by using the web tool pkCSM-pharmacokinetics to predict hepatotoxicity and oral rate acute toxicity LD_50_ values [30]. Predicted LD_50_ values ranged from 2.498 to 3.875 mol/kg. When the liver suffered some damage, the normal metabolism of xenobiotics or drugs was disrupted and could lead to liver failure [35]. The hepatotoxicity descriptor predicted that of the 12 compounds present, this included hepatotoxicity.

Drug-likeness descriptors selected using the Lipinski and Veber rules were calculated with SwissADME. The rule of five by Lipinski argued that good absorption or permeation was more likely when the molecular weight (MW) < 500 Da, equalled the number of hydrogen bond donors (nHbds) < 5, Log P < 5, and number of hydrogen bond acceptors (nHbas) < 10. Two other important descriptors, such as the number of rotatable bonds (nrotb) < 10 and topological polar surface area (PSA) < 140 Å were determined [36]. Analyses of the synthesized compounds **4**–**15** indicated no violations of these rules, except for PSA > 140 Å2, suggesting that all compounds would display well behaved absorption or permeation.

### 2.3. Antiprotozoal In Vitro Activity

The compounds **4***–***15** were tested in vitro against bloodstream trypomastigotes of two T. cruzi strains (NINOA and INC-5) (Table 2). Anti-T. cruzi properties were expressed in terms of percentage lysis of the flagellates for each compound at concentrations of 5, 10, and 50 mg/mL, after 24 h of parasite exposition. Nfx and Bnz were used as reference drugs. In general, compounds **6**–**11** were more active than the reference drugs. First, as can be seen in the data of Table 2, the NINOA strain was more susceptible to compounds **6** and **8**–**11**. The INC-5 strain was more susceptible to compounds **6**, **7**, and **10** than to compounds **4**, **5**, **8**, **9**, and **11**. Compounds **12**–**15** showed no activity towards both strains.

The observed results on the trypomastigotes of T. cruci could be explained in several ways. The most active compounds were those with a 4-hydroxy-3-methoxy substitution pattern in the aromatic ring over the 4-position of the quinoline, but this effect was enhanced when an OMe group was a substitute in the 7-position of the quinoline. However, when the substitution pattern was 3-hydroxy-4-methoxy and there was an OMe group on position-8 there was a slight increase in activity, but in a smaller proportion than that of the most active compound. Other factors considered could be steric or electronic. Compounds **8**–**11** were the most active against T. cruzi (NINOA and INC-5 strains), when the size of the 4-OH group was increased to a benzyloxy group; the new **12**–**15** compounds did not exhibit activity, indicating the influence of the steric factor and the violations of the Veber rules. The amine and nitrile groups, at 2- and 3-position, respectively, were very important for the interaction with its target. The compounds **4** and **8** did not have substitution over the quinoline ring; however, they maintained good activity on the two strains studied. A possible explanation for this could be related to the low values of topological polar surface area (PTSA) and log p.

## 3. Materials and Methods

Thin-layer chromatography was carried out on Merck silica F_254_ 0.255-mm plates, and spots were visualized by UV fluorescence at 254 nm. Elemental analyses were performed using a PerkinElmer 2400 CHN elemental analyzer. The results were within ±0.4% of the predicted values. NMR spectra were obtained using a JEOL Eclipse™ at 270 MHz for ^1^H NMR and at 67.9 MHz for ^13^C NMR using CDCl_3_ or DMSO*d6,* and were reported in ppm downfield from the residual CHCl_3,_ or dimethyl sulfoxide (DMSO) (δ 7.25 or 2.50 for ^1^H NMR and 77.0 or 39.8 for ^13^C NMR, respectively) (see the Supporting Information for the original spectra). The infrared (IR) spectra were recorded on a Shimadzu^TM^ model 470 spectrophotometer using KBr pellets. Diffraction data were measured on a Rigaku AFC-7S diffractometer with a Mercury CCD detector using graphite-monochromated Mo-Kα radiation (λ = 0.71070 Å). A Thomas micro hot-stage device was used to determine the melting points (mp). All organic products or solvents (from Sigma-Aldrich Group, St. Louis, MO, USA) were used directly or distilled and dried in the usual manner, respectively. Compounds **4**–**7** were reported previously [15].

### 3.1. General Procedure for the Synthesis of 2-Amino-5,6-dihydro-4-(phenylsubstituted) benzo[h]quinoline-3-carbonitrile Derivatives ***8***–***15***

Benzaldehyde substituted 1 (1 mmol), malononitrile 2 (1 mmol), 1-tetralone substituted 3 (1 mmol), and ammonium acetate (1.5 mmol), in 10 mL of toluene, with a catalytic amount of acetic acid, were combined in a 20 mL round-bottom flask fitted with a reflux condenser and Dean-Stark trap. The mixture was heated under reflux for 5 h and the solvent was then evaporated using a rotary evaporator. The residue was then poured into crushed ice. The desired product was isolated, filtered, and washed with cold water-ethanol (1:1). The solid was recrystallized from ethanol.

#### 3.1.1. 2-Amino-5,6-dihydro-4-(4-hydroxy-3-methoxyphenyl)benzo[h]quinoline-3-carbonitrile (**8**)

White solid. Yield: 0.319 g. m.p: >300 °C. IR (KBr pellet cm^−1^): 3444, 2976, 2898, 2208, 1603, 1545, 1504, and 1260; ^1^H NMR, CDCl_3_, δ ppm: 2.66-2.77 (m, 4H, and H_5,6_), 3.91 (s, 3H, and OCH_3_), 5.21 (brs, 2H, and NH_2_), 5.79 (brs, 1H, and OH), 6.82–6.83 (m, 2H, and H_2ꞌ_,_6ꞌ_), 7.00 (d, 1H, H_5ꞌ_, and J = 8.7 Hz), 7.25 (m, 1H, and H_7_), 7.30–7.33 (m, 2H, and H_8,9_), 8.25 (m, 1H, and H_10_); ^13^C NMR, DMSOd6, δ ppm: 24.6, 28.0, 56.3, 89.7, 113.4, 115.9, 117.8, 119.0, 121.9, 126.2, 127.2, 128.3, 130.6, 134.1, 139.6, 147.7, 147.9, 153.9, 154.4, and 159.4; Anal. calcd. for C_21_H_17_N_3_O_2_: % C 73.45, H 4.99, and N 12.24. Found: % C 73.43, H 5.01, and N 12.39.

#### 3.1.2. 2-Amino-5,6-dihydro-4-(4-hydroxy-3-methoxyphenyl)7-methoxybenzo[h]quinoline-3-carbonitrile (**9**)

White solid. Yield: 0.336 g. m.p: 182–184 °C. IR (KBr pellet cm^−1^): 3424, 3376, 2976, 2898, 2224, 1616, 1552, 1510, 1424, 1366, and 1261. ^1^H NMR, CDCL_3_, δ ppm: 2.59–2.75(m, 4H, and H_5,6_), 3.84 (s, 3H, and OCH_3_), 3.89 (s, 3H, and OCH_3_), 5.26 (brs, 2H, and NH_2_), 5.81 (brs, 1H, and OH), 6.81–6.83 (m, 2H, and H_2ꞌ_,_6ꞌ_), 6.93 (d, 1H, H_8_, and J = 7.40 Hz), 7.01 (d, 1H, H_5ꞌ_, and J = 8.40 Hz), 7.30 (t, 1H, H_9_, and J_1_ = 7.40 Hz), 7.90 (d, 1H, H_10_, and J = 7.40 Hz). ^13^C NMR, CDCl_3_, δ ppm: 20.2, 24.1, 55.7, 56.2, 90.7, 111.3, 112.3, 114.7, 117.3, 118.5, 120.6, 122.1, 127.4, 127.6, 127.9, 134.5, 145.4, 146.5, 146.6, 156.2, and 157.9. Anal. calcd. for C_22_H_19_N_3_O_3_: % C 70.76, H 5.13, and N 11.25. Found: % C 70.80, H 5.15, and N 11.34.

#### 3.1.3. 2-Amino-5,6-dihydro-4-(4-hydroxy-3-methoxyphenyl)8-methoxybenzo[h]quinoline-3-carbonitrile (**10**)

White solid. Yield: 0339 g. m.p. 188–190 °C. IR (KBr pellet cm^−1^): 3360, 2976, 2898, 2219, 1603, 1542, 1436, 1356, and 1248. ^1^H NMR, CDCL_3_, δ ppm: 2.66–2.74 (m, 4H, and H_5_,_6_), 3.84 (s, 3H, and OCH_3_), 3.90 (s, 3H, and OCH_3_), 5.31 (brs, 2H, and NH_2_), 5.84 (brs, 1H, and OH), 6.71 (d, 1H, H_7_, and J = 2.70 Hz), 6.82–6.83 (m, 2H, and H_2′,__6′_), 6.90 (dd, 1H, H_9_, J_1_ = 2.50 Hz, and J_2_ = 8.70 Hz), 7.02 (d, 1H, H_5ꞌ_, J_1_ = 8.40 Hz, and J_2_ = 8.40 Hz), 8.22 (d, 1H, H_10_, and J = 8.70 Hz); 13C NMR, DMSOd6, δ ppm: 24.6, 28.4, 55.8, 56.3, 88.7, 113.2, 113.1, 113.4, 115.9, 118.0, 121.9, 126, 127.4, 128.0, 141.7, 147.6, 147.9, 153.5, 154.6,159.4, and 161.4. Anal. calcd. for C_22_H_19_N_3_O_3_: % C 70.76, H 5.13, and N 11.25. Found: % C 70.72, H 5.11, and N 11.37.

#### 3.1.4. 2-Amino-5,6-dihydro-4-(4-hydroxy-3-methoxyphenyl)9-methoxybenzo[h]quinoline-3-carbonitrile (**11**)

White solid. Yield: 0.332 g. m.p. 224–226 °C. IR (KBr pellet cm^−1^): 3360, 2976, 2898, 2224, 1612, 1542, 1507, and 1260. ^1^H NMR, CDCL_3_, δ ppm: 2.65-269 (m, 4H, and H_5_,_6_), 3.91 (s, 3H, and OCH_3_), 3.92 (s, 3H, and OCH_3_), 5.52 (brs, 2H, and NH_2_), 5.80 (brs, 1H, and OH), 6.81–6.84 (m, 2H, and H_2′_,_6′_), 6.90 (dd, 1H, H_8_, J_1_ = 2.70 Hz, and J_2_ = 8.20 Hz), 7.01 (d, 1H, H_5′_, and J = 8.40 Hz), 7.09 (d, 1H, H_7_, and J = 8.40 Hz), 7.85 (d, 1H, H_10_, and J = 2.70 Hz); 13C NMR, DMSOd6, δ ppm: 24.9, 27.2, 55.8, 56.3, 89.9, 110.8, 113.4, 115.9, 116.7, 117.7, 119.2, 121.9, 127.3, 129.4, 131.8, 135.1, 147.7, 147.9, 153.9, 154.2, 158,8, and 159.4. Anal. calcd. for C_22_H_19_N_3_O_3_: % C 70.76, H 5.13, and N 11.25. Found: % C 70.76, H 5.14, and N 11.31.

#### 3.1.5. 2-Amino-4-[4-(benzyloxy)-3-methoxyphenyl]5,6-dihydrobenzo[h]quinoline-3-carbonitrile (**12**)

White solid. Yield. 0.359 g. m.p. 228–229 °C. IR (KBr pellet cm^−1^): 3344, 2976, 2898, 2192, 1619, 1536, 1504, 1244. ^1^H NMR, DMSO d6, δ ppm: 2.57–2.74 (m, 4H, H_5,6_), 3.78 (s, 3H, OCH_3_), 5.14 (s, 2H, OCH_2_), 6.70 (s, 2H, NH_2_), 6.90 (dd, 1H, H_6′_, J_1_ = 2.2 Hz, J_2_ = 8.4 Hz), 7.01 (s, 1H, H_2′_), 7.16 (d, 1H, H_5′_, J = 8.4 Hz), 7.20–7.50 (m, 8H, Ar), 8.16–8.19 (m, 1H, H_10_); ^13^C NMR, DMSOd6, δ ppm: 24.6, 27.9, 56.3, 70.5, 89.7, 113.2, 113.6, 117.7, 119.0, 121.6, 126.2, 127.3, 128.4, 128.5, 129.0, 130.6, 134.1, 137.5, 139.6, 148.7, 149.3, 153,6, 154.5, 159.4. Anal. calcd. for C_28_H_23_N_3_O_2_: % C 77.58, H 5.35, N 9.69. Found: % C 77.61, H 5.37, N 9.82.

#### 3.1.6. 2-Amino-4-[4-(benzyloxy)-3-methoxyphenyl]5,6-dihydro-7-methoxybenzo[h]quinoline-3-carbonitrile (**13**)

White solid. Yield: 0.403 g. m.p. 180–181 °C. IR (KBr pellet cm^−1^): 3328, 2898, 2192, 2208, 1603, 1542, 1500, 1452, 1414, 1360, 1312, and 1251. ^1^H NMR, DMSO d6, δ ppm: 2.53–2.69 (m, 4H, and H_5,6_), 3.78 (s, 3H, and OCH_3_), 3.81 (s, 3H, and OCH_3_), 5.14 (s, 2H, and OCH_2_), 6.67 (brs, 2H, and NH_2_), 6.90 (dd, 1H, H_6′_, J_1_ = 2.2 Hz, and J_2_ = 8.4 Hz), 7.00 (s, 1H, and H_2′_), 7.08 (d, 1H, H_8_, and J = 7.9 Hz), 7.16 (d, 1H, H_5′_, and J = 8.4 Hz), 7.30–7.51 (m, 6H, and Ar), 7.82 (d, 1H, H_10_, and J = 7.9 Hz); ^13^C NMR, DMSOd6, δ ppm: 20.4, 24.0, 56.1, 56.3, 70.5, 89.7, 112.8, 113.2, 113.6, 117.7, 118.5, 118.8, 121.6, 127.5, 127.6, 128.5, 129.0, 135.1, 137.5, 148.7, 149.3, 153,5, 154.5, 156.3, and 159.3. Anal. calcd. for C_29_H_25_N_3_O_3_: % C 75.14, H 5.44, and N 9.07. Found: % C 75.12, H 5.46, and N 9.12.

#### 3.1.7. 2-Amino-4-[4-(benzyloxy)-3-methoxyphenyl]5,6-dihydro-8-methoxybenzo[h]quinoline-3-carbonitrile (**14**)

White solid. Yield: 0.370 g. m.p. 231–232 °C. IR (KBr pellet cm^−1^): 3328, 2898, 2192, 2208, 1619, 1539, 1504, 1248, and 1235. ^1^H NMR, DMSO d6, δ ppm: 2.55–2.54 (m, 4H, and H_5,6_), 3.79 (s, 3H, and OCH_3_), 3.81 (s, 3H, and OCH_3_), 5.15 (s, 2H, and OCH_2_), 6.63 (brs, 2H, and NH_2_), 6.89–6.95 (m, 2H, and H_2´6´_), 7.00 (d, 1H, H_7_, and J = 1.7 Hz), 7.18 (d, 1H, H_5´_, and J = 8.4 Hz), 7.30-7.48 (m, 6H, and Ar), 8.12 (d, 1H, H_10_, and J = 8.7 Hz); ^13^C NMR, DMSOd6, δ ppm: 24.6, 28.4, 55.8, 56.3, 70.5, 89.7, 113.2, 113.2, 117.9, 121.5, 121.6, 126.2, 127.3, 128.5, 128.7, 129.0, 130.6, 134.1, 137.6, 141.7, 148.7, 149.3, 153.1, 154.6, and 159.4. Anal. calcd. for C_29_H_25_N_3_O_3_: % C 75.14, H 5.44, and N 9.07. Found: % C 75.09, H 5.44, and N 9.31.

#### 3.1.8. 2-Amino-4-[4-(benzyloxy)-3-methoxyphenyl]5,6-dihydro-9-methoxybenzo[h]quinoline-3-carbonitrile (**15**)

White solid. Yield: 0.389 g. m.p. 228–229 °C. IR (KBr pellet cm^−1^): 3360, 2898, 2224, 2192, 1616, 1548, 1510, 1260, and 1232. ^1^H NMR, DMSO d6, δ ppm: 2.49–2.67 (m, 4H, and H_5,6_), 3.78 (s, 3H, and OCH_3_), 3.80 (s, 3H, and OCH_3_), 5.14 (s, 2H, and OCH_2_), 6.70 (brs, 2H, and NH_2_), 6.89–7.01 (m, 3H, and H_2′6′,7_), 7.16 (d, 1H, H_5´_, and J = 8.4 Hz), 7.35–7.47 (m, 6H, and Ar), 7.72 (d, 1H, H_10_, and J = 2.7 Hz); ^13^C NMR, DMSOd6, δ ppm: 24.9, 27.1, 55.8, 56.3, 70.5, 89.8, 110.9, 113.2, 113.6, 116.7, 117.6, 119.2, 121.6, 128.5, 129.0, 129.1, 129.4, 131.8, 135.1, 137.5, 148.7, 149.3, 153.6, 154.3, 158.8, and 159.4. Anal. calcd. for C_29_H_25_N_3_O_3_: % C 75.14, H 5.44, and N 9.07. Found: % C 75.19, H 5.47, and N 9.23.

### 3.2. X-ray Analysis on Compound ***6***

Crystals of Compound 6 suitable for X-ray diffraction were obtained by slow evaporation of a solution in ethanol. The structure was solved by direct methods and refined on F^2^ by full-matrix least-squares, using all reflections and weights w = [**¦Ò**^2^(F_o_^2^) + (a P)^2^ + b P]**^−^**^1^, with P = (F_o_^2^ + 2 F_c_^2^)/3. The C-bonded H atoms were placed in calculated positions and refined using a riding atom model with fixed C-H distances (0.93 Å for CH and CH_2_, 0.96 Å for CH_3_), and with U_iso_ = p U_eq_(parent atom) (p = 1.2 for CH and CH_2_, 1.5 for CH_3_). The N- and O-bonded H atoms were located in difference Fourier syntheses and refined without restraints in the positions and with U_iso_ = p U_eq_(parent atom) (p = 1.2 for N, 1.5 for O). Due to its proximity to a special position (0, y, ¼; Wyckoff site 4e), the ethanol molecule of solvation did not refine satisfactorily; it was assigned fixed coordinates and s.o.f = 0.50, and a refined common U_iso_. The C-bonded H atoms were treated as indicated above, while the O-bonded H atom could not be located. The following computer programs were used: data collection, data reduction, cell refinement and absorption correction, CRYSTALCLEAR [37]; structure solution, SHELXS-97 [38]; structure refinement, SHELXL-97 [38]; geometrical calculations, PLATON [39]; molecular graphics, and ORTEP-3 [40]. The structure solution, the refinement, and the drawings were carried out with the aid of the WinGX [41] suite of programs. The crystallographic.cif file containing data for Compound 6 was deposited at Cambridge Crystallographic Data Center (CCDC no. 794547). Data could be obtained free of charge via https://www.ccdc.cam.ac.uk/structures/ (accessed on 30 September 2021).

Crystal Data for C_22_H_19_N_3_O_3_ ^.^ 0.5 C_2_H_5_O (MW = 403.84 g/mol): monoclinic, space group C2/c (No. 15), a = 16.361(4) Å, b = 7.8450(16) Å, c = 31.540(7) Å, β = 101.128(4)°, V = 3972.1(15) Å3, Z = 8, T = 295(2) K, μ(CuKα) = 0.091 mm^−1^, Dcalc = 1.326 g/cm^3^, F(000) = 1672; crystal size 0.28 × 0.013 × 0.09 mm^3^, 23189 reflections measured (2.5°–27.7°), and 3916 unique (Rint = 0.0994). The final R1 was 0.0753 (I > 2σ(I)) and wR2 was 0.1533 (all data), and GOOF = 1.039 (all data).

### 3.3. Antiprotozoal In Vitro Activity

Two Mexican strains of *T. cruzi* (INC-5 and NINOA) were used. The parasites were maintained in the laboratory in the vector Triatoma pallidipennis (Insecta: Hemiptera) and by successive passages in NIH female mice of 25–30 g. INC-5 strain was obtained from a chronic case of Chagas disease in the city of Guanajuato, Mexico; NINOA strain was obtained from an acute case of Chagas disease by xenodiagnosis in the city of Oaxaca, Mexico [42]. The test was evaluated in vitro on bloodstream trypomastigotes of both strains. Blood was obtained by cardiac puncture of female NIH mice at the peak of parasitemia (4 × 10^6^ parasites/mL). Heparin was used as an anticoagulant. Infected blood was diluted with a 0.85% sterile saline solution to a final concentration of 1 × 10^6^ trypomastigotes/mL. For each compound, the stock was prepared in DMSO (10 mg/mL). Then, each stock solution was serially diluted in sterile distilled water to the desired concentration. For the in vitro assay, sterile 96-well culture plates were used. Each plate contained 195 mL of the suspension of bloodstream trypomastigotes and 5 mL of each dilution, so that each compound was evaluated at a final concentration of 5, 10, and 50 mg/mL. As a control, we used a final concentration of DMSO 1%, since it had been found that concentrations <5% had no impact on mobility, infectivity, and ultrastructural morphology. Nfx and Bzn were used as reference compounds. The concentrations of each compound were evaluated in triplicate. The plates were incubated at 4 °C for 24 h. The trypanocidal activity was evaluated by counting the number of bloodstream trypomastigotes after the incubation time by the Brener method [43,44,45]. Briefly, 5 mL of blood were placed on slides, covered with a coverslip, and the flagellates were examined with an optical microscope at 40× magnification. The trypanocidal activity was expressed in terms of lysis of the flagellates after incubation time compared with the control group.

## 4. Conclusions

Thus, in summary, we have synthesized through the sequential multicomponent reaction of aromatic aldehydes, malononitrile, and 1-tetralone in the presence of ammonium acetate and acetic acid, in a high yield manner, a series of methoxybenzo[*h*]quinoline-3-carbonitrile analogs that exhibited attractive antichagasic activities. A study in silico on absorption, distribution, metabolism, excretion, and toxicity (ADME/Tox) profiling to help to describe the molecular properties related to the pharmacokinetic aspects in the human body of these compounds was reported. These compounds represented an excellent opportunity for the development of simple, cheap and active species for the treatment of Chagas disease in its acute stage. The 4-hydroxy-3-methoxy phenyl substitution patterns at position-4 of the quinoline ring improved the antitrypanosomal activity of these compounds. The activity was affected when the substitution pattern in the phenyl group at position-4 of quinoline changes to 3-hydroxy-4-methoxy. This study showed the importance of methoxybenzo[*h*]quinoline-3-carbonitrile derivatives as potential anticancer and anti-Chagas agents. Experiments aimed to shed further light are currently under study in our laboratory and the results will be disclosed in due time.

## Figures and Tables

**Table 1:** In silico evaluation of physicochemical properties of compounds **4**–**15**.

No	Log P	MW	Hba	Hbd	Rotb	Viol	LogSw	%HIA	FU	CLtot	LD_50_
**4**	3.29	343.4	4	2	2	0	−4.218	97.90	0.034	0.469	2.518
**5**	3.30	373.4	5	2	3	0	−4.384	99.55	0.059	0.506	2.507
**6**	3.28	373.4	5	2	3	0	−4.078	100	0.086	0.434	2.558
**7**	3.27	373.4	5	2	3	0	−4.104	100	0.102	0.448	2.543
**8**	3.27	343.4	4	2	2	0	−4.229	94.79	0.040	0.458	2.514
**9**	3.29	373.4	5	2	3	0	−4.304	96.43	0.067	0.495	2.522
**10**	3.28	373.4	5	2	3	0	−4.184	100	0.077	0.437	2.498
**11**	3.27	373.4	5	2	3	0	−4.187	100	0.095	0.451	2.504
**12**	4.84	433.5	5	1	5	0	−5.217	97.69	0.210	0.530	3.875
**13**	4.83	463.5	6	1	6	0	−4.736	98.56	0.253	0.570	3.119
**14**	4.84	463.5	6	1	6	0	−5.079	96.95	0.225	0.465	3.096
**15**	4.82	463.5	6	1	6	0	−4.893	96.19	0.240	0.514	3.177

Log P, partition coefficient; MW, molecular weight; Hba, Hydrogen bond acceptors; Hbd, Hydrogen bond donors; Rotb, rotatable bonds; Viol, Lipinski’s violations; LogSw: Water Solubility; %HIA, human intestinal absorption; P; FU, fraction unbound; CLtot, total clearance; LD_50_, oral rate acute toxicity; TPSA: topological polar surface area >140 Å2 for **5**–**7**, **9**–**15**.

**Table 2:** In vitro activity of **4**–**15** against bloodstream trypomastigote of T. cruzi.

	*Trypanosoma cruzi*			*Trypanosoma cruzi*		
	NINOA Strain LC (mg/mL)			INC-5 Strain LC (mg/mL)		
No.	5	10	50	5	10	50
**Nfx**	18.3	31	37.5	0	20	30
**Bnz**	23.3	33.6	35.9	0	5	15
**4**	28.7	29.7	29.9	0	10	17
**5**	22	25.8	32.9	21	22	27
**6**	36.5	37.9	45	20	30	36
**7**	15.4	22	23	21.6	25.6	28.2
**8**	35.5	39.2	39.9	20.1	21.1	28
**9**	42.1	44.2	37.5	10	27	30
**10**	33.3	33.5	18.3	24	33	28
**11**	31.3	35.7	28.6	4	13.7	14.6
**12**	0	0	0	0	0	0
**13**	0	0	0	0	0	0
**14**	0	0	14	0	0	0
**15**	0	0	0	0	0	0

LC: lytic concentration; n: 3 determinations. Trypanocidal activity was expressed in terms of percentage lysis of the flagellates.

## Data Availability

The data presented in this study are contained within the article and Supplementary Materials.

## Supplementary Materials

The following are available online: ^1^H/^13^C NMR for compounds **8**, **10**, **11**, and ^1^H/^13^C NMR, DEPT 135°, and HETCOR spectra for compounds **13**–**15**.

## Sample Availability

Samples of the compounds are available from the authors.
