# Supplementary material for: One-Pot Multicomponent Synthesis of Methoxybenzo[h]quinoline-3-carbonitrile Derivatives; Anti-Chagas, X-ray, and In Silico ADME/Tox Profiling Studies
Source: Molecules. 2021 Nov 19;26(22):6977. doi: 10.3390/molecules26226977 (PMC8619670; doi:10.3390/molecules26226977)

Supplementary Material for

One-pot multicomponent synthesis of methoxybenzo[*h*]quinoline-3-carbonitrile derivatives. Anti-Chagas, x-ray, and *in silico* ADME/Tox profiling studies.

Hegira Ramírez [1,2] *, Katiuska Charris [1], Esteban Fernandez-Moreira [3], Benjamín Nogueda-Torres [4], Mario V. Capparelli [5], Jorge Ángel [6], Jaime Charris [1]*

[1]Laboratorio de Síntesis Orgánica, Facultad de Farmacia, Universidad Central de Venezuela, Apartado 47206, Los Chaguaramos, 1041-A Caracas, Venezuela.
[2]Facultad de Medicina, Universidad de Las Américas, 170503 Quito, Ecuador.
[3]Escuela de Medicina, Universidad Espíritu Santo, 092301 Samborondón, Ecuador.
[4]Escuela Nacional de Ciencias Biológicas, Departamento de Parasitología, Instituto Politécnico Nacional, México, CDMX 11340, México.
[5]Unidad de Estructura Molecular, Fundación Instituto de Estudios Avanzados (IDEA), Apartado 17606, Caracas 1015-A, Venezuela.
[6]Laboratorio de Síntesis Orgánica y Diseño de Fármacos, Dpto. de Química, Facultad Experimental de Ciencias, Universidad del Zulia, Maracaibo, Venezuela.

Correspondence: jaime.charris@ucv.ve; hegira.ramirez@udla.edu.ec. Tel.: +58-412-2359228 JC, +593-97-8706334 HR.

Table of Contents

		Page
S1	The ^{1}H NMR spectrum of **8**	3
S2	The ^{13}C NMR spectrum of **8**	4
S3	The ^{1}H NMR spectrum of **10**	5
S4	The ^{13}C NMR spectrum of **10**	6
S5	The ^{1}H NMR spectrum of **11**	7
S6	The ^{13}C NMR spectrum of **11**	8
S7	The ^{1}H NMR spectrum of **13**	9
S8	The ^{13}C NMR spectrum of **13**	10
S9	The DEPT 135° spectrum of **13**	11
S10	The HETCOR spectrum of **13**	12
S11	The ^{1}H NMR spectrum of **14**	13
S12	The ^{13}C NMR spectrum of **14**	14
S13	The DEPT 135° spectrum of **14**	15
S14	The HETCOR spectrum of **14**	16
S15	The ^{1}H NMR spectrum of **15**	17
S16	The ^{13}C NMR spectrum of **15**	18

S1 The ^{1}H NMR spectrum of **8**

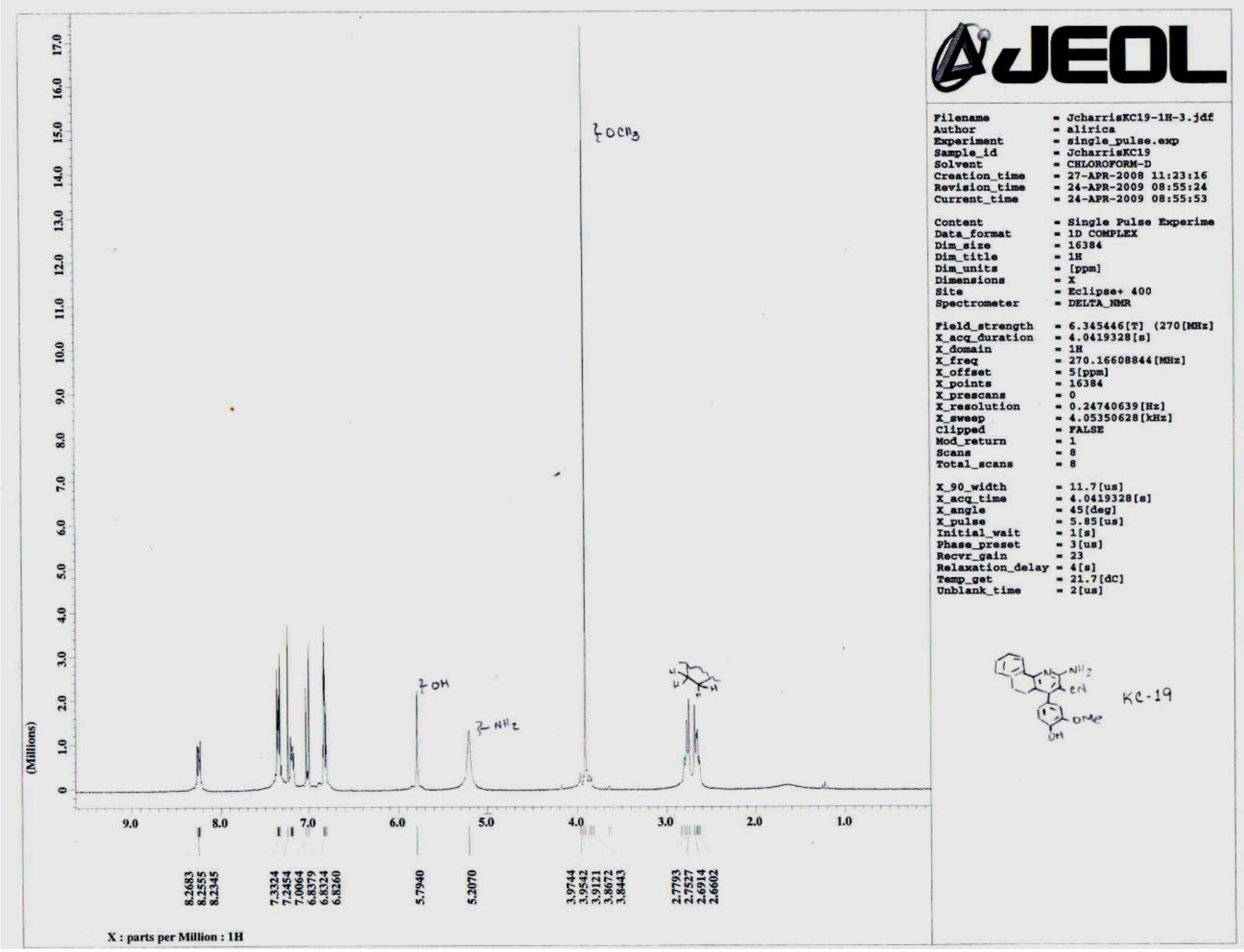

S2 The ^{13}C NMR spectrum of **8**

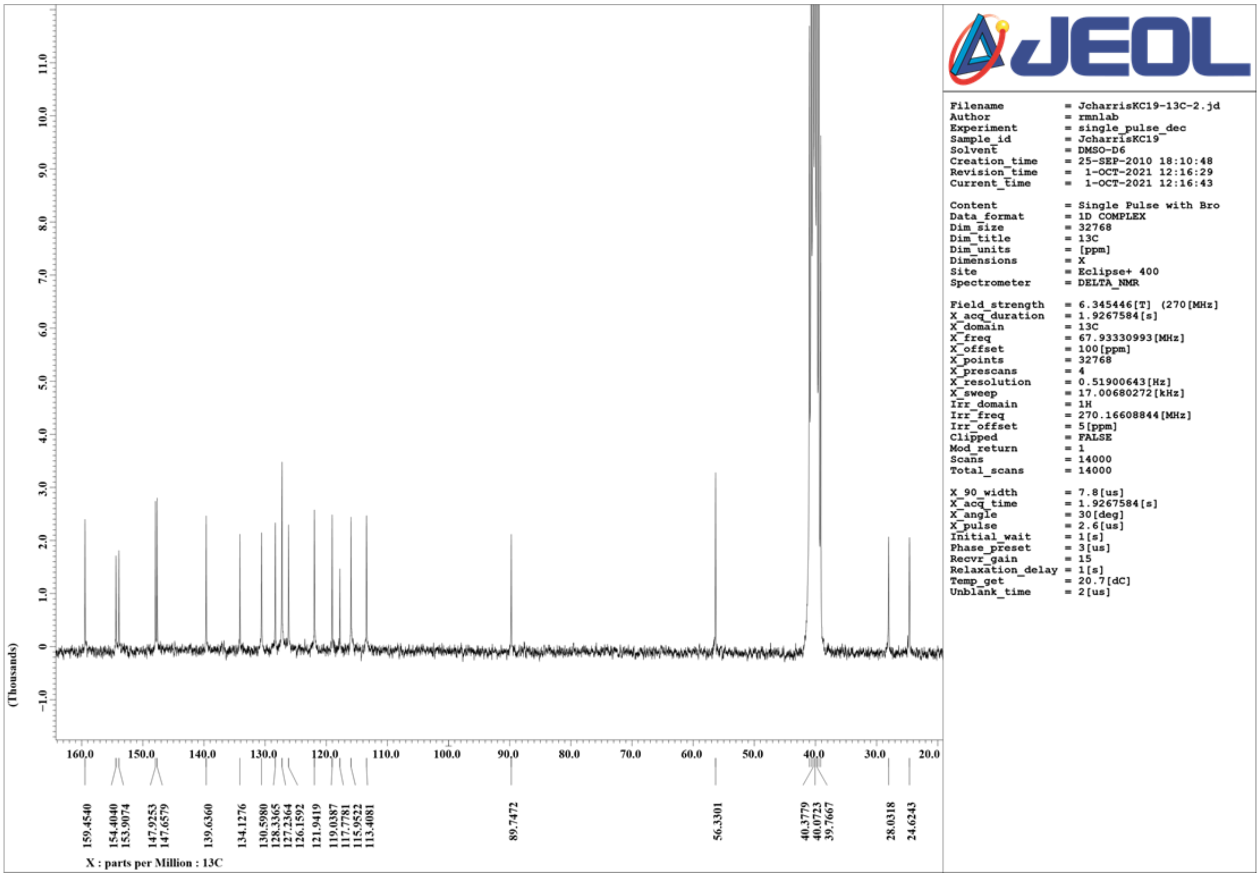

S3 The ^{1}H NMR spectrum of **10**

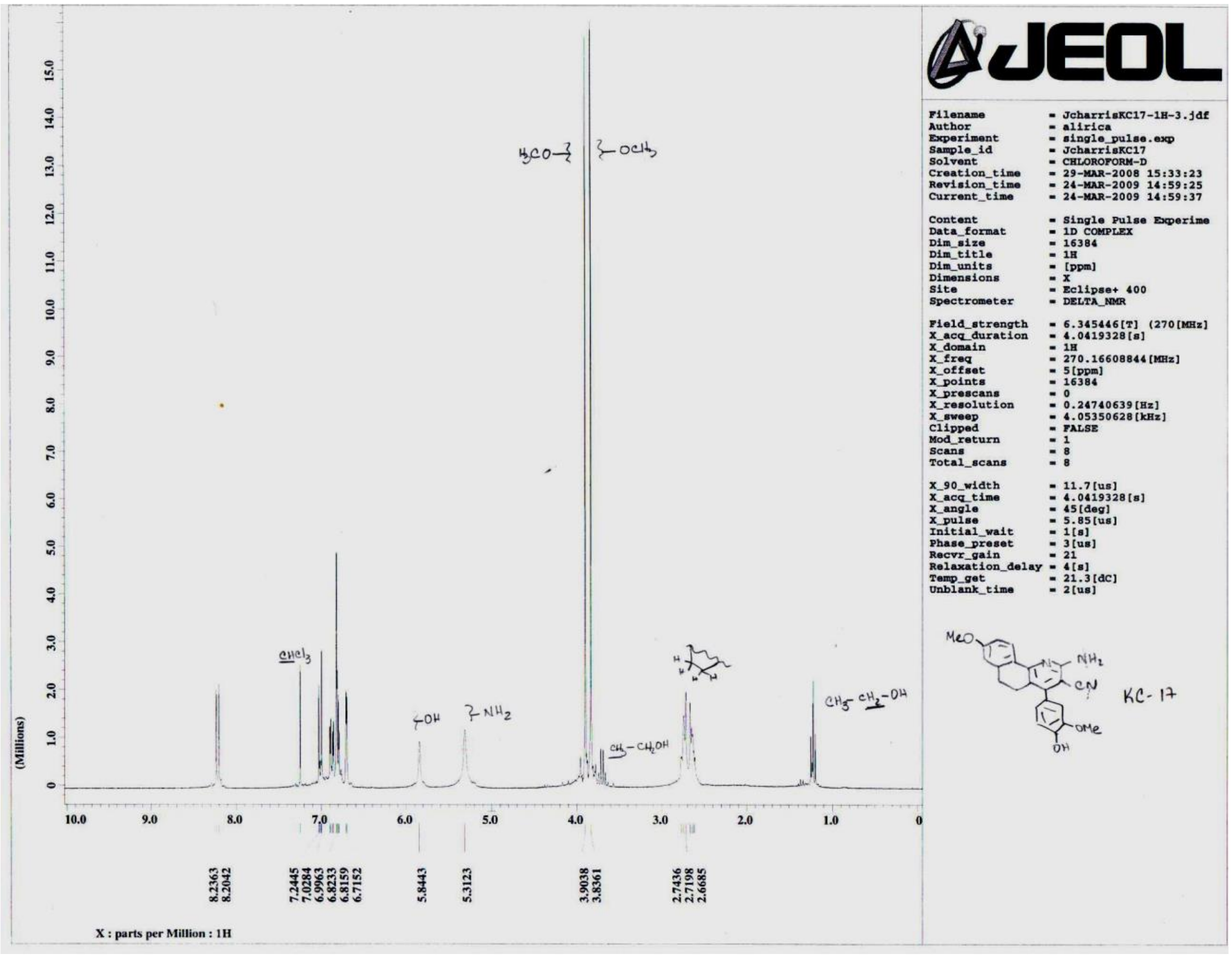

S4 The ^{13}C NMR spectrum of **10**

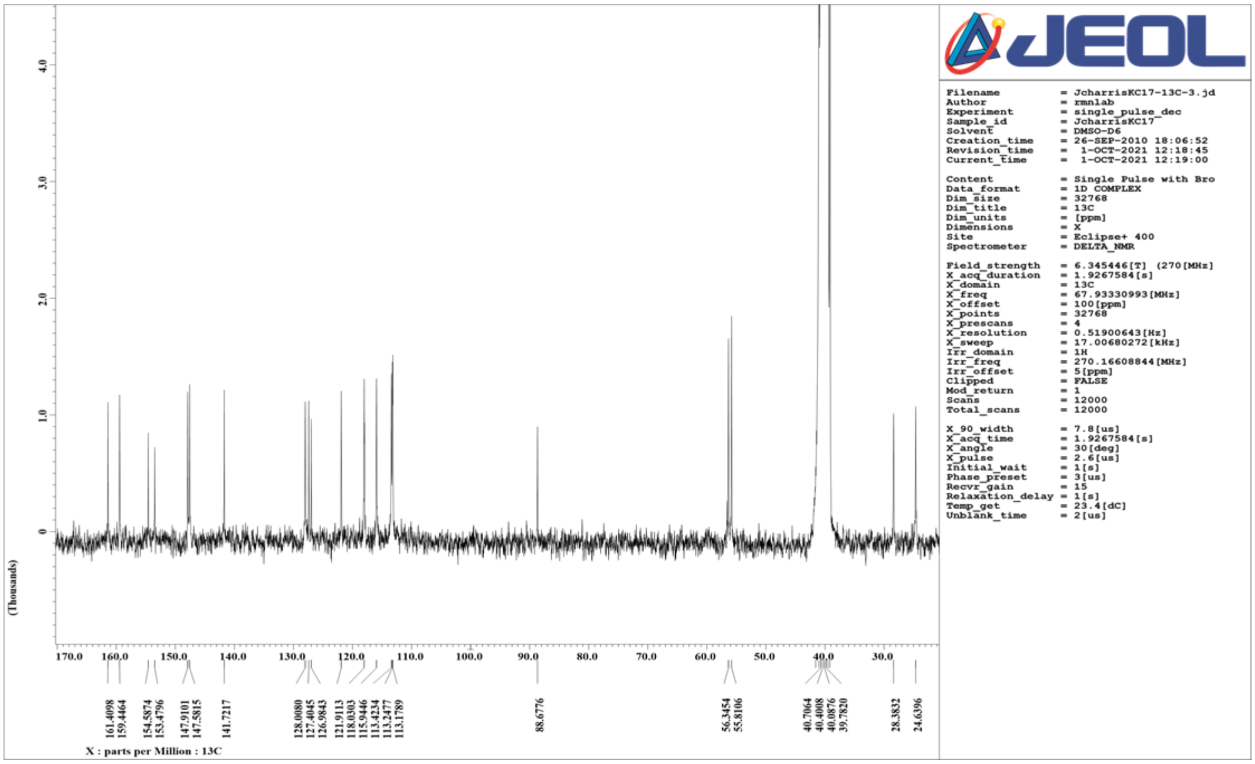

S5 The ^{1}H NMR spectrum of **11**

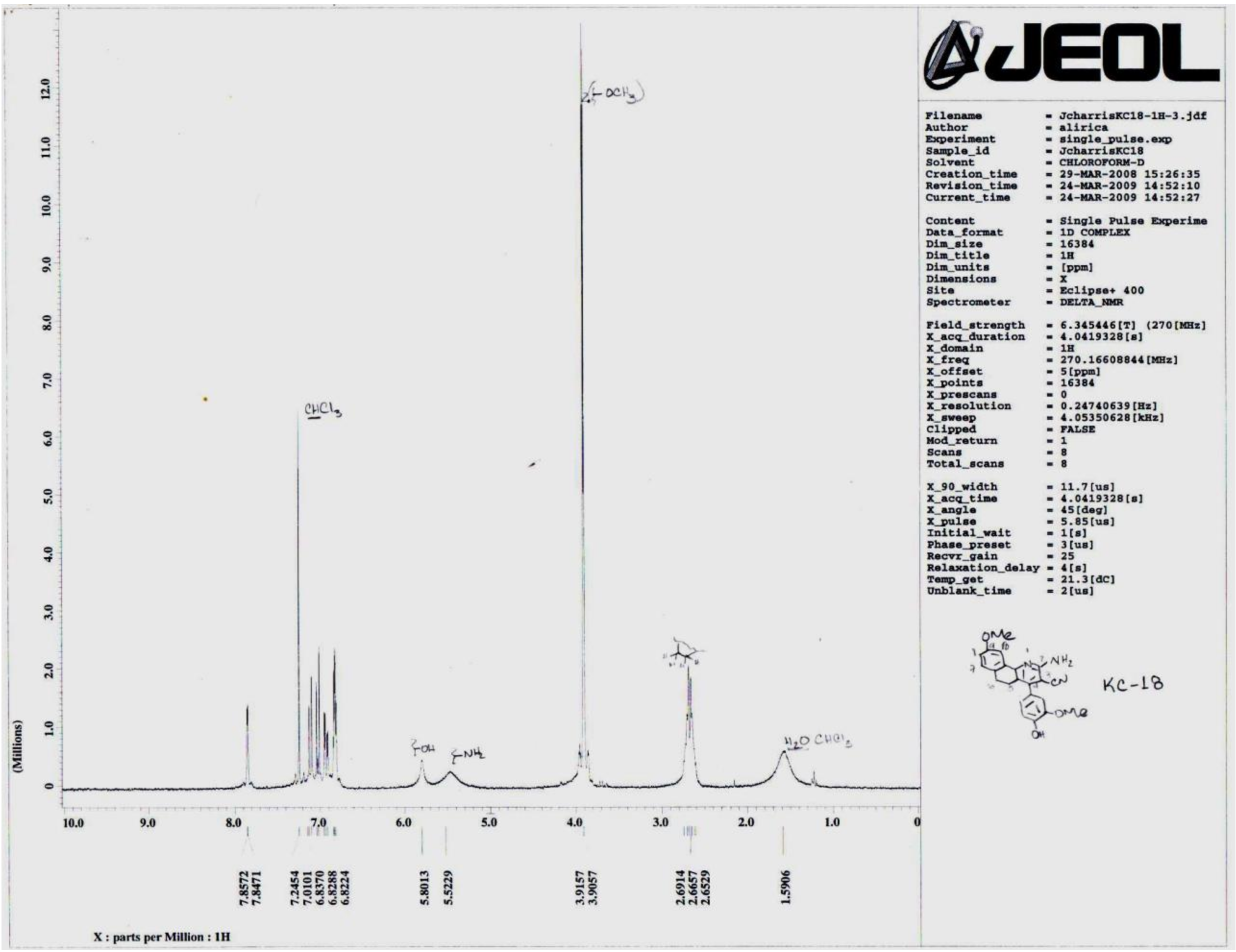

S6 The ^{13}C NMR spectrum of **11**

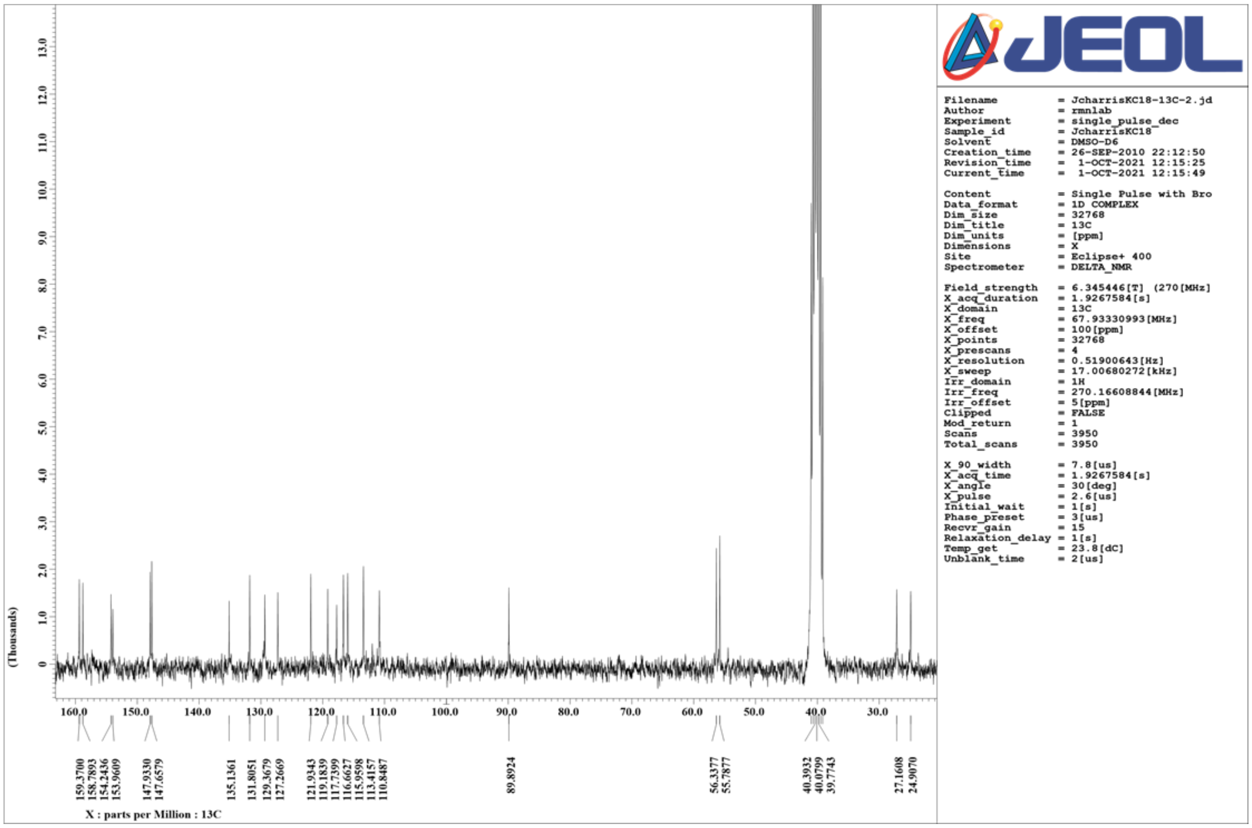

S7 The ^{1}H NMR spectrum of **13**

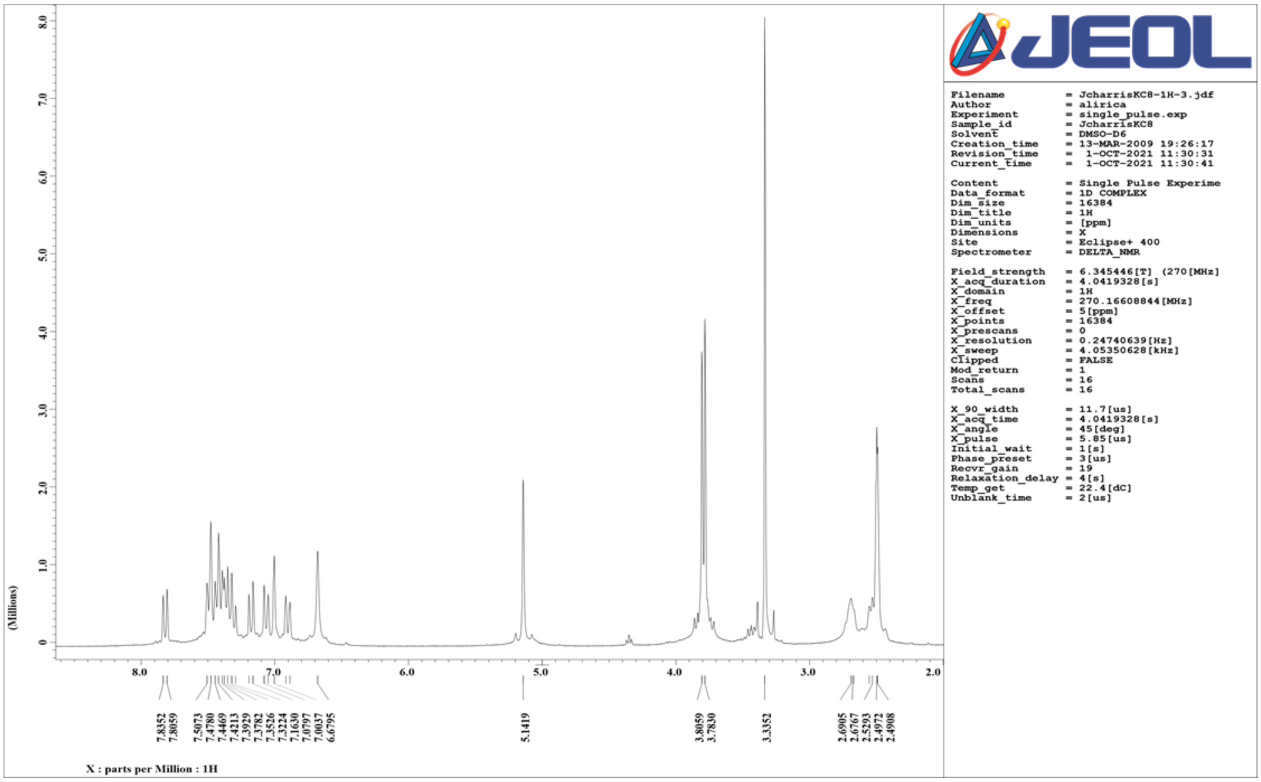

S8 The ^{13}C NMR spectrum of **13**

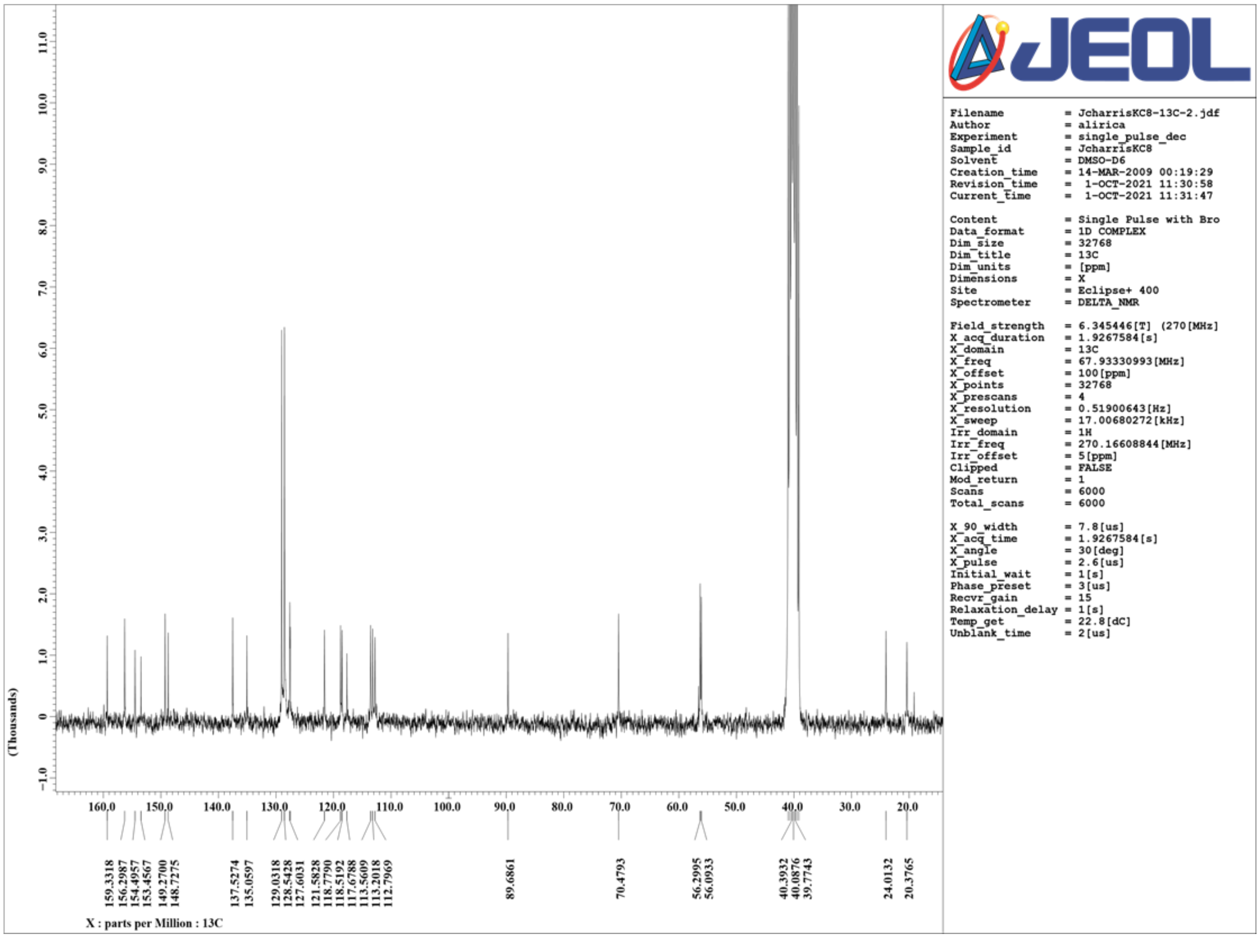

S9 The DEPT 135° spectrum of **13**

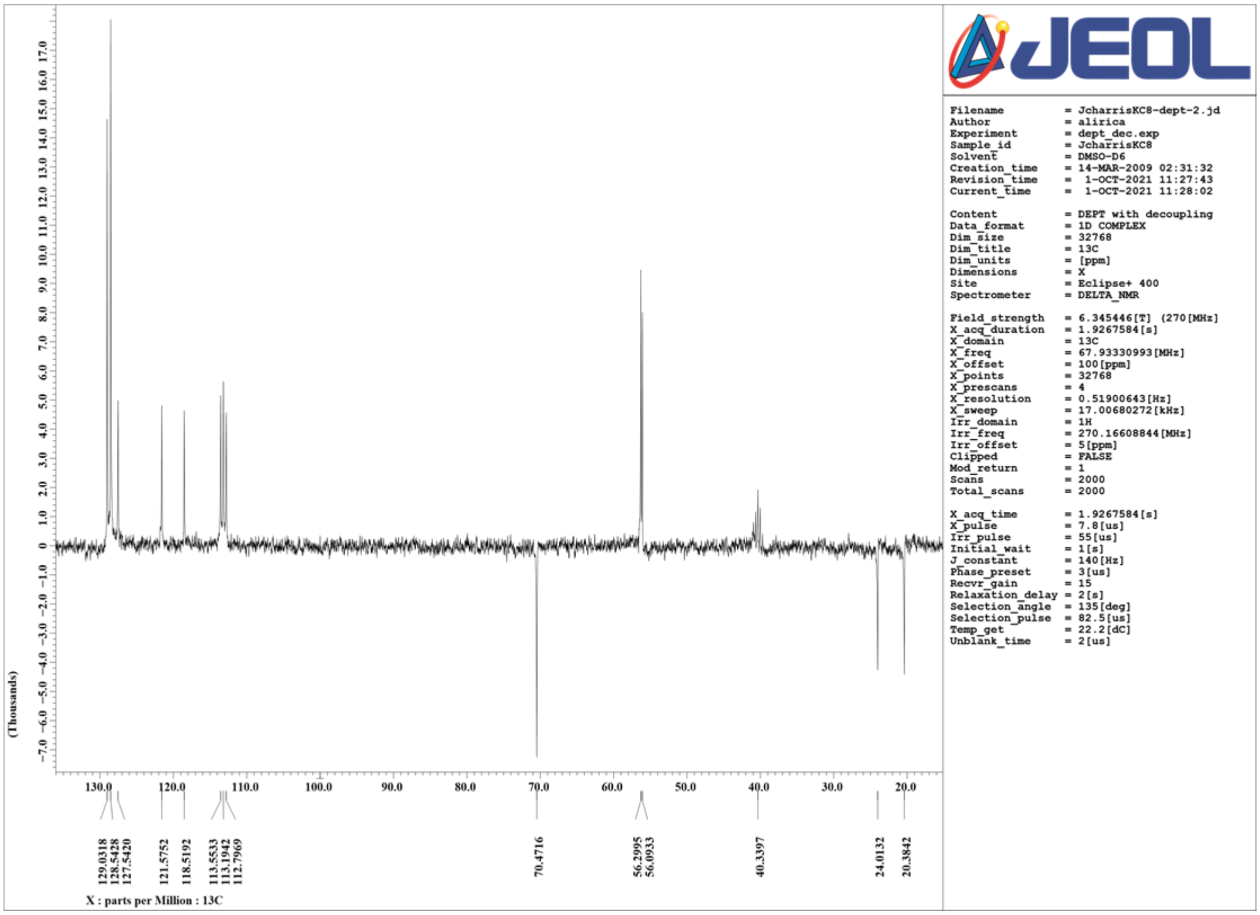

S10 The HETCOR spectrum of **13**

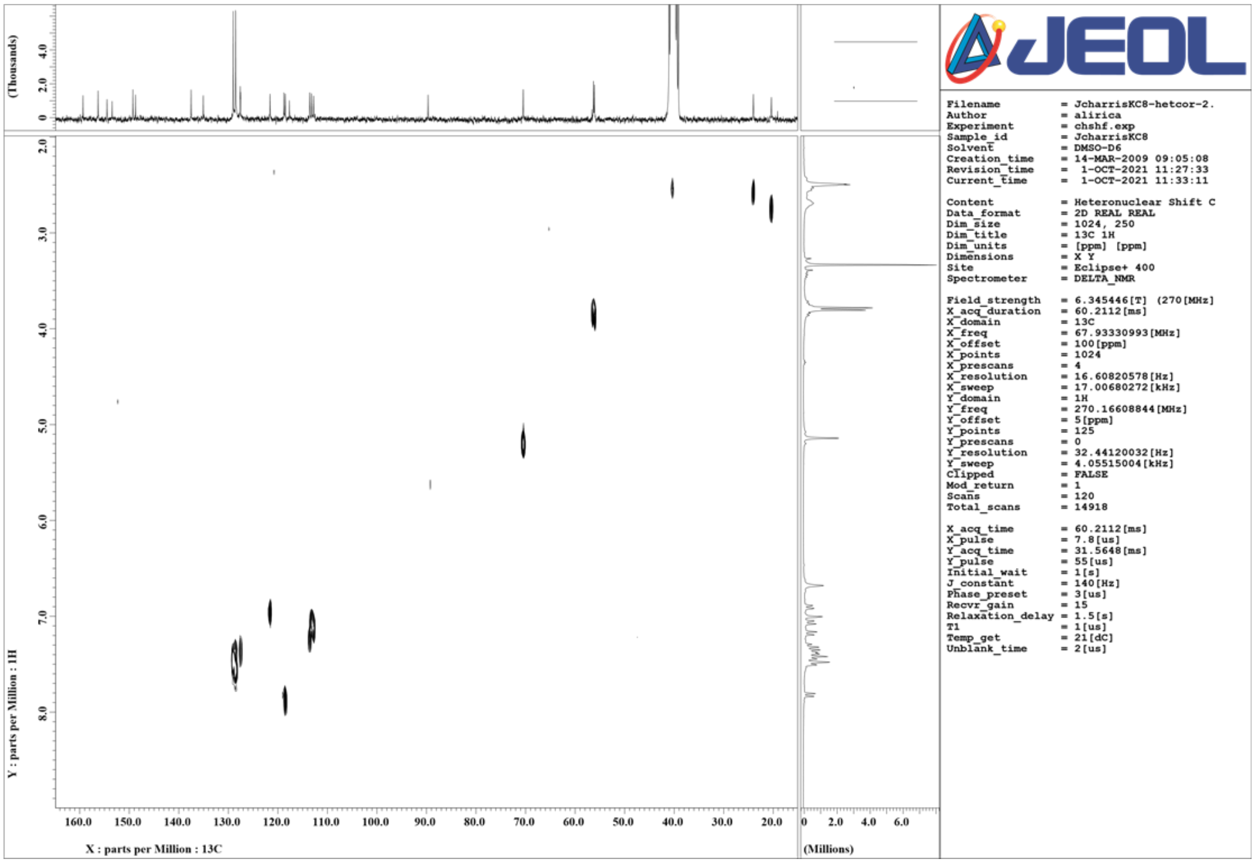

S11 The ^{1}H NMR spectrum of **14**

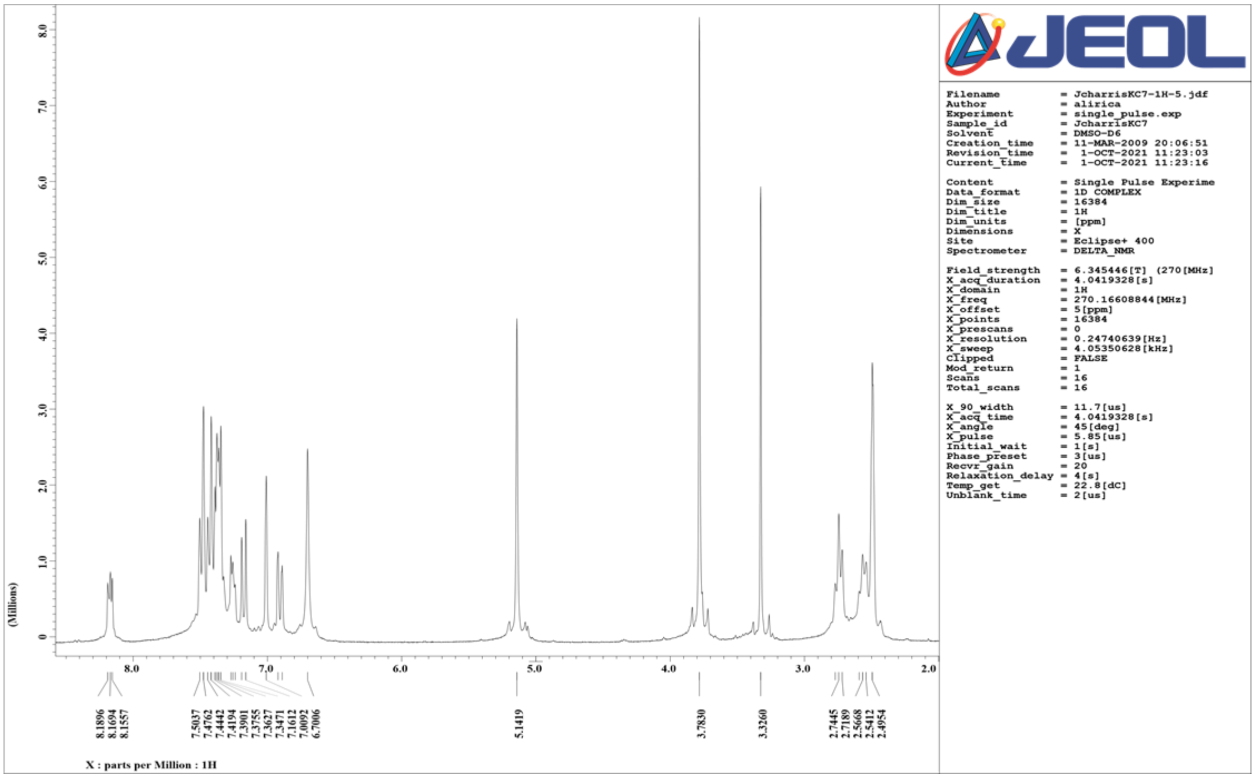

S12 The ^{13}C NMR spectrum of **14**

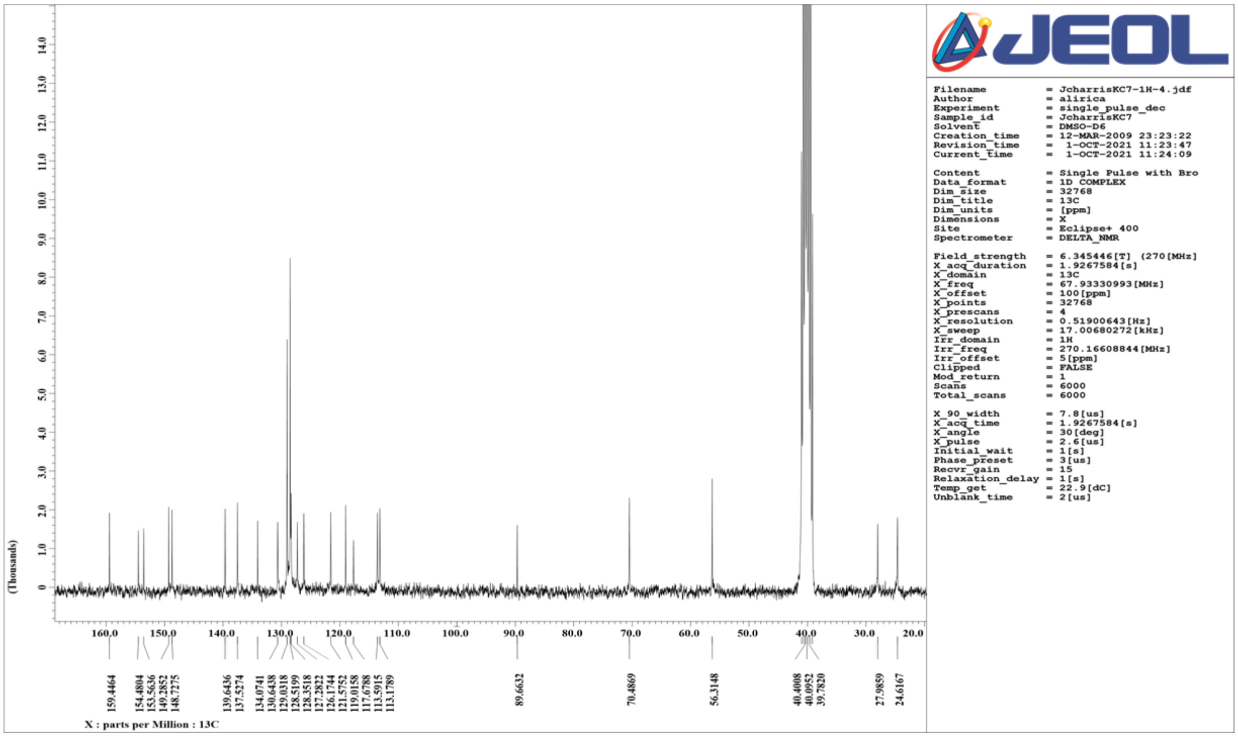

S13 The DEPT 135° spectrum of **14**

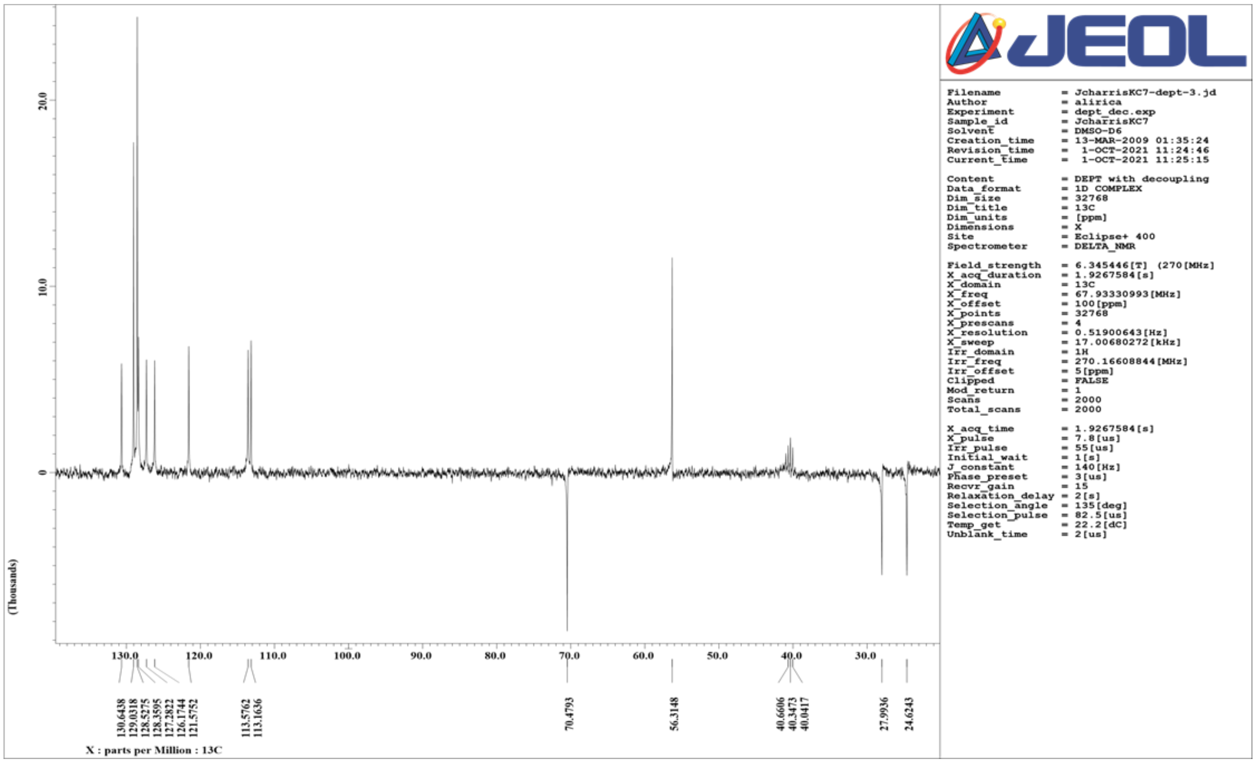

S14 The HETCOR spectrum of **14**

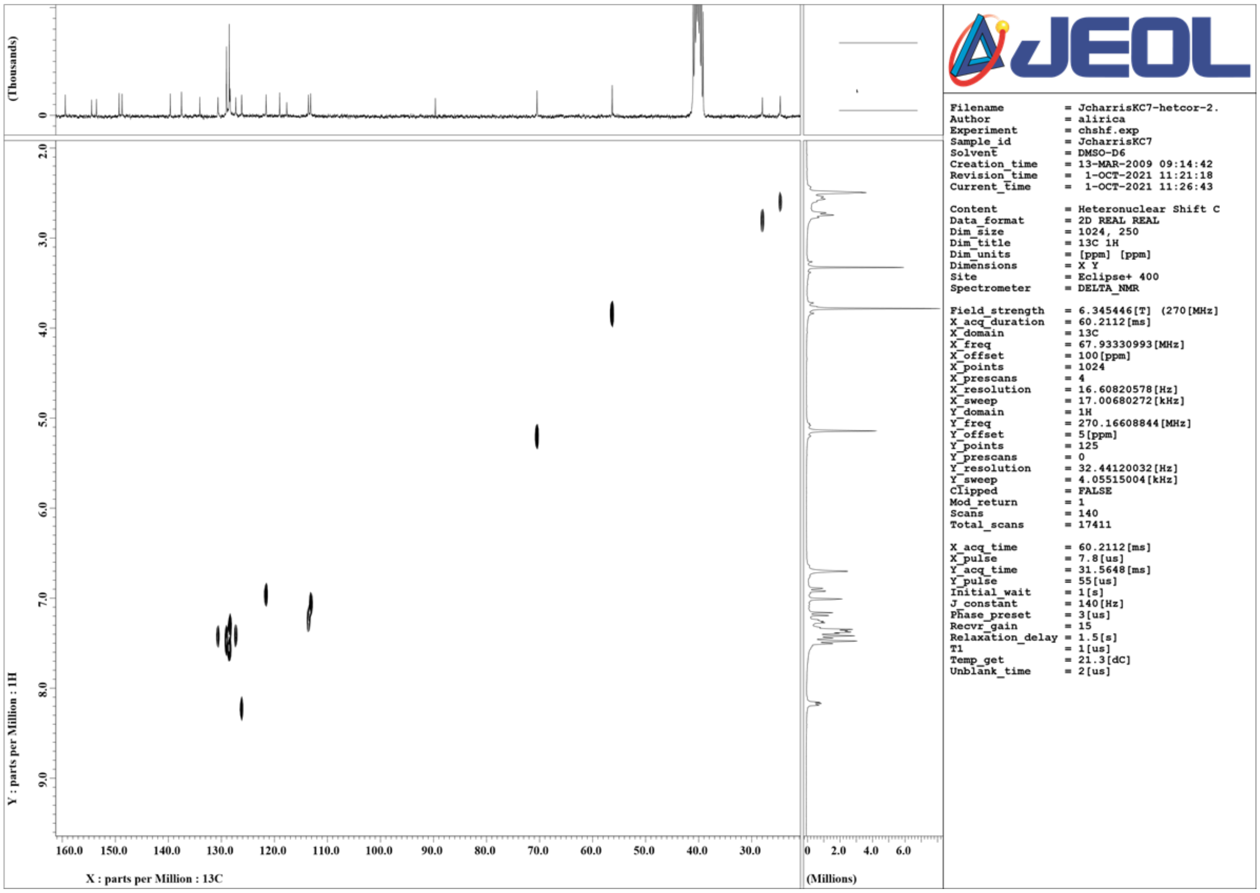

S15 The ^{1}H NMR spectrum of **15**

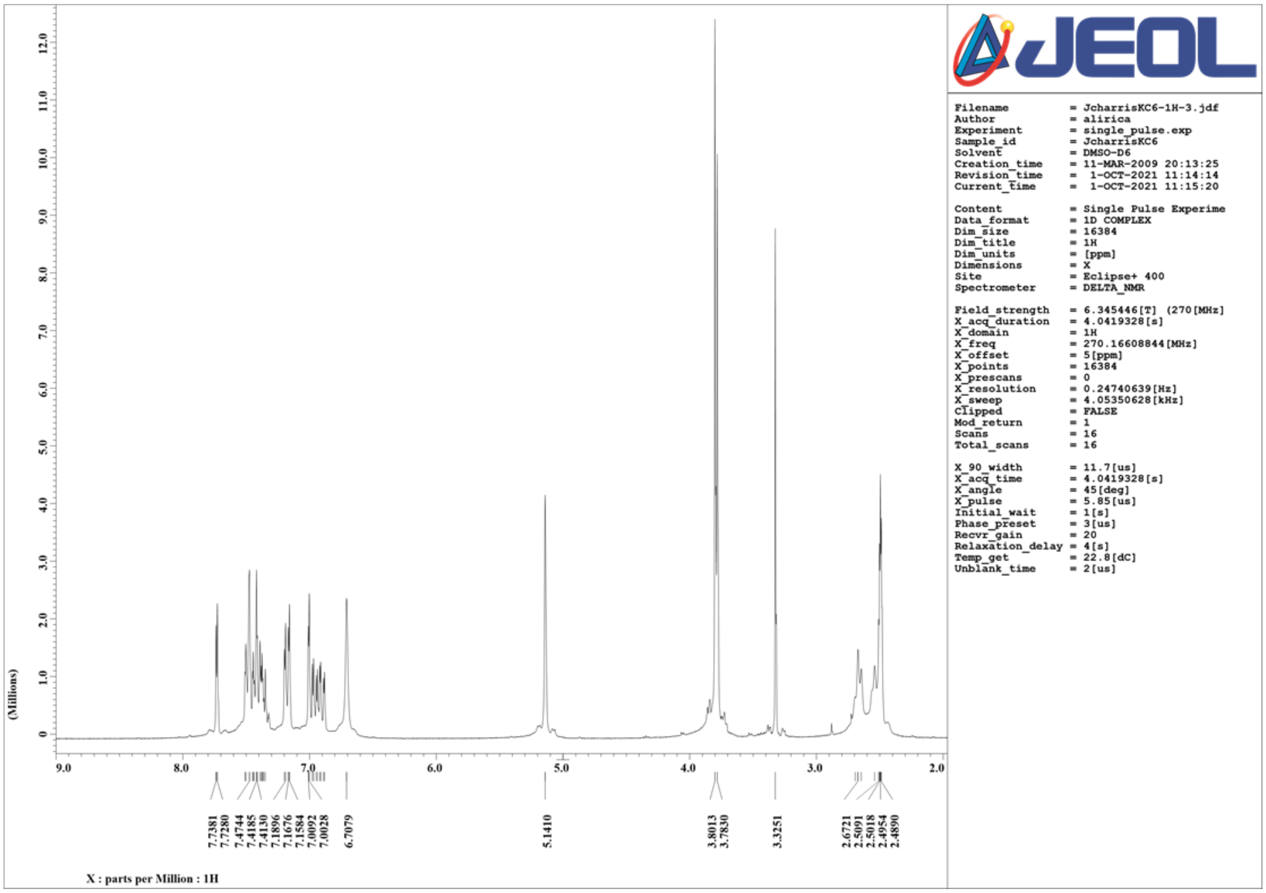

S16 The ^{13}C NMR spectrum of **15**

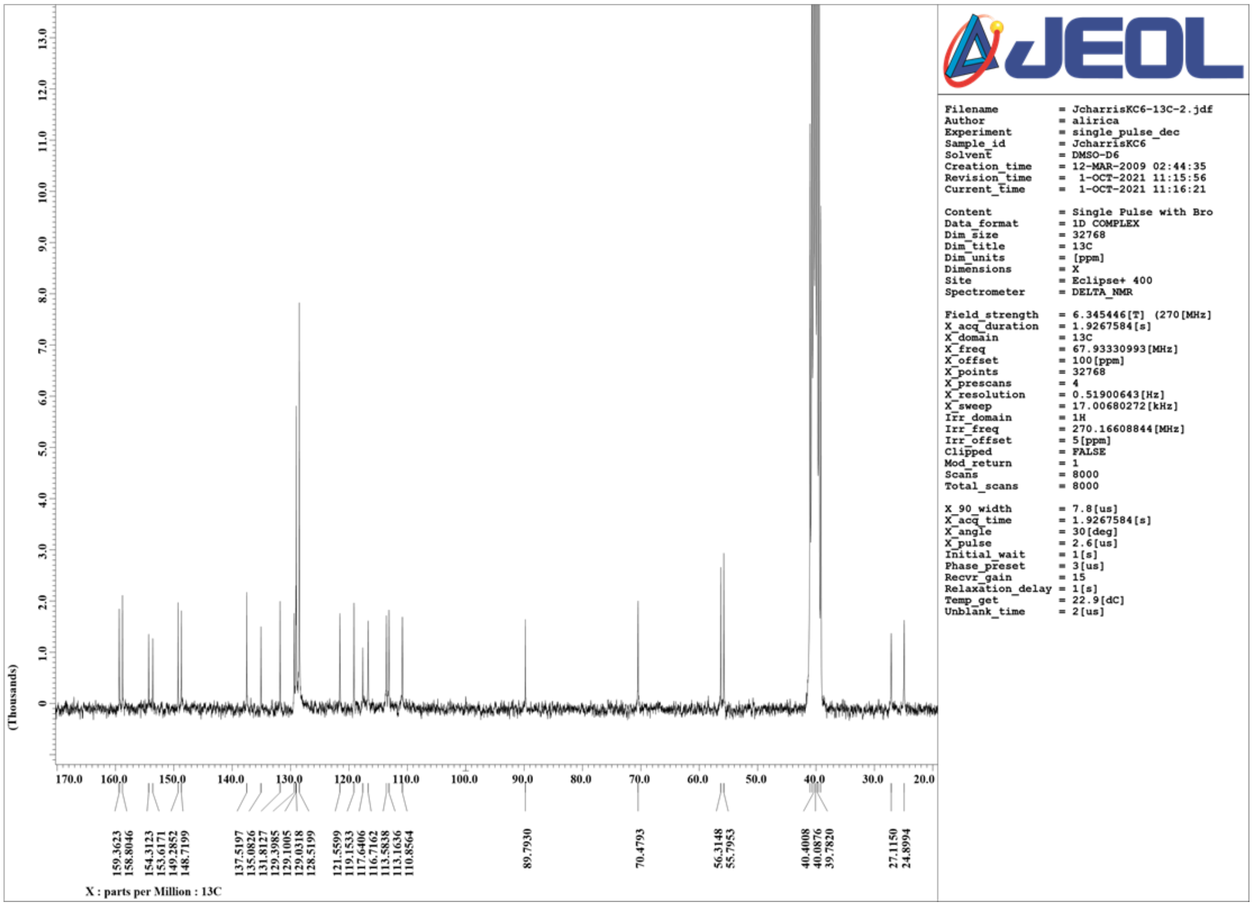

Supplement: Supplementary file 1 [file molecules-26-06977-s001.zip › molecules-1452033-supplementary.pdf]
